# Advanced primary urethral cancer: a case report

**DOI:** 10.1186/s13256-019-2253-y

**Published:** 2019-11-29

**Authors:** Huan Chen, Li Li Zou, Chuan Jiang Dong, Tao Li, Zi Qiang Dong

**Affiliations:** 1 Yichang Central People’s Hospital, Institute of Urology, The First College of Clinical Medical Science, Three Gorges University, No. 183, Xiling District, Yichang, 443000 Yichang Hubei Province China; 2 The Institute of Infection and Inflammation, China Three Gorges University, No. 8 University road, Xiling District, Yichang, 443000 Yichang Hubei Province China

**Keywords:** Urethral cancer, Diagnose, Surgery, Intravesical chemotherapy

## Abstract

**Background:**

Primary urethral cancer is exceedingly rare, resulting in a limitation in clinicians’ experience, and an accurate diagnosis is often delayed due to the non-specific clinical presentation. Here, we present this case report to show the treatment of a patient with primary urethral cancer. Our patient was diagnosed as having primary urethral cancer in the First Clinical Hospital of Yichang by cystoscopy and biopsy. Due to her age, poor physical tolerance, and economic condition, she refused radical operation. Since there is no definite guideline for the treatment process of primary urethral cancer in clinics, operation methods and postoperative adjuvant treatments vary in different hospitals, leading to diverse prognostic effects.

**Case presentation:**

An 88-year-old Asian woman had difficulty in urinating for more than 6 months and the syndrome was aggravated for 1 month. She chose a relatively conservative treatment plan: primary tumor resection combined with bladder perfusion chemotherapy. Postoperative pathology revealed “urethra” high-grade urothelial carcinoma (sarcoma-like variants) with extensive necrosis. After treatment with intravesical chemotherapeutic drug (hydroxycamptothecin 40 mg), she was eventually released from our hospital in a stable condition. Postoperation follow-up was performed to observe to what extent this conservative treatment plan improved the quality of life and overall survival time of our patient.

**Conclusions:**

She needed radical resection according to the actual situation. However, her age restricted her tolerance to general anesthesia; relatively conservative treatment options are available to ensure a high quality of life. The treatment of primary tumor resection combined with bladder perfusion chemotherapy is feasible. This case highlights the importance of the dissemination of new cases and optimizing primary urethral cancer diagnosis to obtain an effective treatment.

## Background

The European Association of Urology (EAU) defines a tumor with its very first lesion located in the urethra as primary urethral cancer (PUC) [1]. It is a very aggressive malignant tumor with high invasiveness both in men and women [2, 3], which accounts for less than 1% of all malignant tumors and 5% of the malignant tumors of the urinary system [4]. However, with social development and the destruction of the environment, the prevalence of the disease has shown an upward tendency. Its onset is related to many factors, such as race and gender, and morbidity increases with age [5]. For males, long-term chronic stimulation (such as repeated catheterization), urethral stricture, post-radiotherapy, and sexually transmitted disease (STD) are the main causes. In female patients, the etiology is quite different from males; the etiology is mainly related to urinary tract infection, urethral diverticulum, and human papilloma virus infection [6], which could greatly increase the morbidity.

Urothelial carcinoma (UC) is the most common histologic type in males, accounting for 47–73% of PUC; squamous cell carcinoma accounts for 12–30% and adenocarcinoma accounts for 5–16% [7–9]. Although UC is also the most common histologic type in females, accounting for approximately 45% of PUC, adenocarcinoma is more common than squamous cell carcinoma, at 29% and 19%, respectively [10]. Derksen *et al*. also verified that adenocarcinoma was the most severe malignancy, followed by squamous cell carcinoma and transitional epithelial cancer [10]. This could be explained by the reason that adenocarcinoma is more likely to occur as a lymph node metastasis, leading to disease progression [10].

The early diagnosis of PUC is crucial for clinical treatment, since this cancer has no obvious symptoms at the first stage and lacks specific screening indicators. At the middle and late stages, there will be some obvious symptoms, such as voiding disorder, difficulty in sexual intercourse, irritation symptoms, hematuria, and so on. There are various types of examinations to diagnose PUC, such as magnetic resonance imaging (MRI) and computed tomography (CT), among which cystoscopy and biopsy are the most accurate ones. Considering the low morbidity of this cancer, there are no unified standards for PUC management and treatment, and there are different treatments of different histologic types of PUC in different hospitals.

Since PUC is rare, the relationship between clinical treatment and prognosis is not clear and there is a lack of corresponding medical records. There are many puzzles in its clinical treatment, such as: If a radical operation is performed on elderly patients with chronic physical failure, can they tolerate the operation? If patients undergo conservative surgery, will their quality of life and life cycle be improved? Can radiochemotherapy be used in the treatment of this disease? These puzzles need to be further explored through clinical practice. Therefore, optimizing the treatment of PUC has become one of the central issues. International health care institutions also hope to improve the efficacy of cancer treatment and the quality of life of patients with PUC [1]. Thus this case report highlights the importance of the dissemination of new cases and optimizing PUC treatment.

## Case presentation

Our patient is an 88-year-old Asian woman who had difficulty in urinating for more than 6 months and the syndrome was aggravated for 1 month. At the same time, urination was accompanied by lower abdominal pain. Urine could be discharged when she forced it to. She had no symptoms concerning urinary frequency and urgency. She had no back pain, and no obvious abnormalities in diet and stools. Before she went to our hospital, she did not perform any special treatments; her physical condition was not very well. She had no history of cardiopulmonary disease, hypertension and diabetes, or cerebrovascular accidents. She had a history of infectious diseases without hepatitis, tuberculosis, or drug allergy. She denied a history of mental illness and she denied a history of surgery, trauma, or blood transfusion. In addition, according to the requirements of the local epidemic prevention department, she underwent complete vaccination progress. Her job is long-term farming. She has a son. She lives in the countryside and the living conditions are not very good. She does not smoke tobacco or consume alcohol.

Physical examination: she was clear-minded, her physiological reflex was normal and the pathological reflex was not elicited. Her pulse was 72 times per minute, blood pressure was 135/80, and body temperature was 36.5 ºC. Her bilateral ureters had no percussion pain. She had no renal tenderness in the double kidney area, and had normal bowel sounds. In the lithotomy position, there was a dark red neoplasm that pointed to 5 o’clock direction. It was approximately 2 × 1 cm in size that grew to the urethra. The surface of the neoplasm was smooth and the boundary was clear, and pus and fur could be seen. Her neoplasm had pressing pain, and accompanied bleeding when it was touched. Her inguinal lymph nodes were not swollen.

After admission to the First Clinical Hospital of Yichang, a relevant auxiliary examination of our patient was performed, and the test results were as follows: Blood Routine Test (Blood-Rt) – hematocrit (HCT) 30.9%, hemoglobin (HGB) 96 g/L, platelets (PLT) 160 × 10^9^/L, red blood cells (RBC) 3.51 × 10^12^/L, and white blood cells (WBC) 7.22 × 10^9^/L. Liver and renal functions – blood urea nitrogen (BUN) 4.90 mmol/L, carbon dioxide binding capacity 20.1 mmol/l, creatinine (CREA) 61.3 umol/L, Ca 2.07 mmol/L, CL 112.8 mmol/L, K 4.34 mmol/L, Na 145.2 mmol/L, alanine aminotransferase (ALT) 6 U/L, and aspartate aminotransferase (AST) 16 U/L. Urine Routine Test (Urine-Rt) – Bacteria (BACT) 2233.70/ul, bilirubin (BIL) – umol/L, occult blood (BLD) + 2 mg/L, ketone (KET) – mmol/L, nitrite (NIT) + 2, protein (PRO) – g/L, RBC 462.80 cells/ul, and Urbilinogen (URO)- normal umol/L.

The chest X-ray results were normal. The kidney and ureter CT results were: our patient had small hepatic cysts and cystic foci in the right adnexa area; her kidneys and ureter showed no obvious abnormal density shadows.

The cystoscopy findings: there was a dark red neoplasm outside our patient’s urethral orifice, in the right rear with percussion pain. Local infiltration anesthesia of the urethra was performed, and the bladder mucosa was still smooth under the observation of 70 degree cystoscope. There were a large number of floating floccules in the inside and no obvious abnormality in the bilateral ureteral opening. The urethral neoplasm extended to the bladder neck with irregular surface and bleeding. The bladder floccules and neoplasms outside the urethra were then collected for biopsy.

The results of pathological examination: the specimens were blood clots and sphacelus, containing a large number of chronic and acute invasive inflammatory cells and a small amount of degenerative abnormal cells, which indicated that it might be a malignant tumor.

**Fig. 1:**
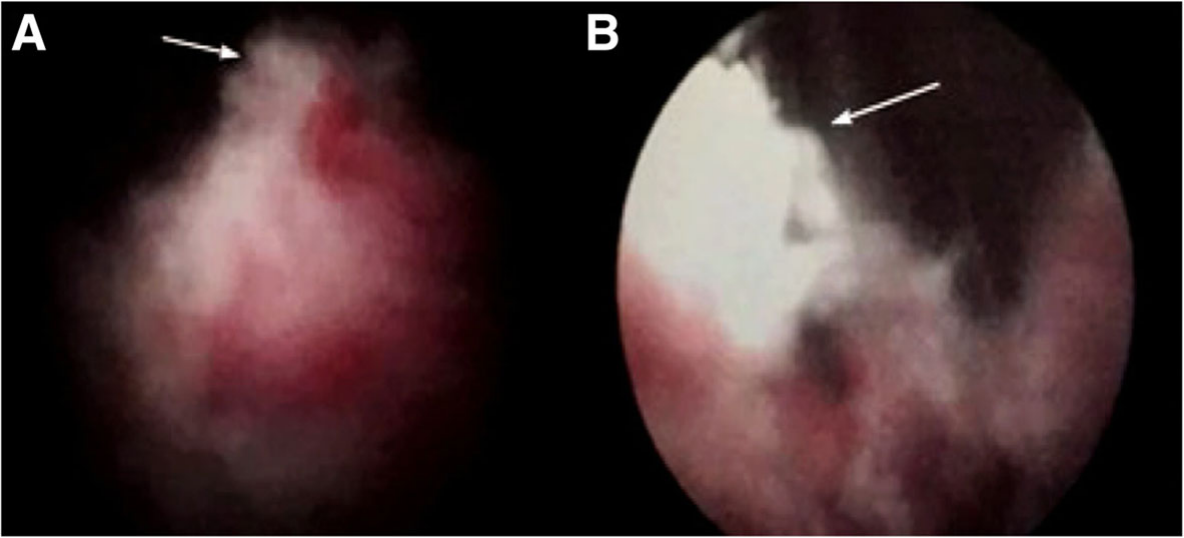
Cystoscopy performed in April 2015. The neoplasm is located in the posterior urethra (arrow) (**a**). It extends beyond the urethra (arrow) (**b**) into the bladder neck and is dark red, easily bleeding. The doctor took a biopsy. (magnification, × 5)

Diagnostic electrocision of urethral mass: after successful subdural anesthesia, our patient took the lithotomy position and the operative area was disinfected with Iodophor (iodine complexed with a solubilizing agent). The operator inserted a 22.5F Karl Storz resectoscope into her bladder. The bladder was observed and the operator found congested trigone of the bladder, which might have been the result of catheterization. The operator used the resectoscope in a retrograde manner to reach the neck of her bladder, which showed the direction of 3–5 points of neoplasm protruding into the bladder, and 6–9 points direction of new neoplasms growing into the urethra and extending to the external urethral orifice. The new neoplasms were crispy and easy to peel and contained visible bleeding. The operator cut surface necrosis with an electric cutting ring, and electrocoagulation was used to stop bleeding followed with flushing out of the specimens. After examination of the complete urethra without obvious residue, an indwelling 20^#^ catheter was inserted; the operator gave local mild compression and connected the catheter with physiological water to flush the bladder (Fig. 1).

**Fig. 2:**
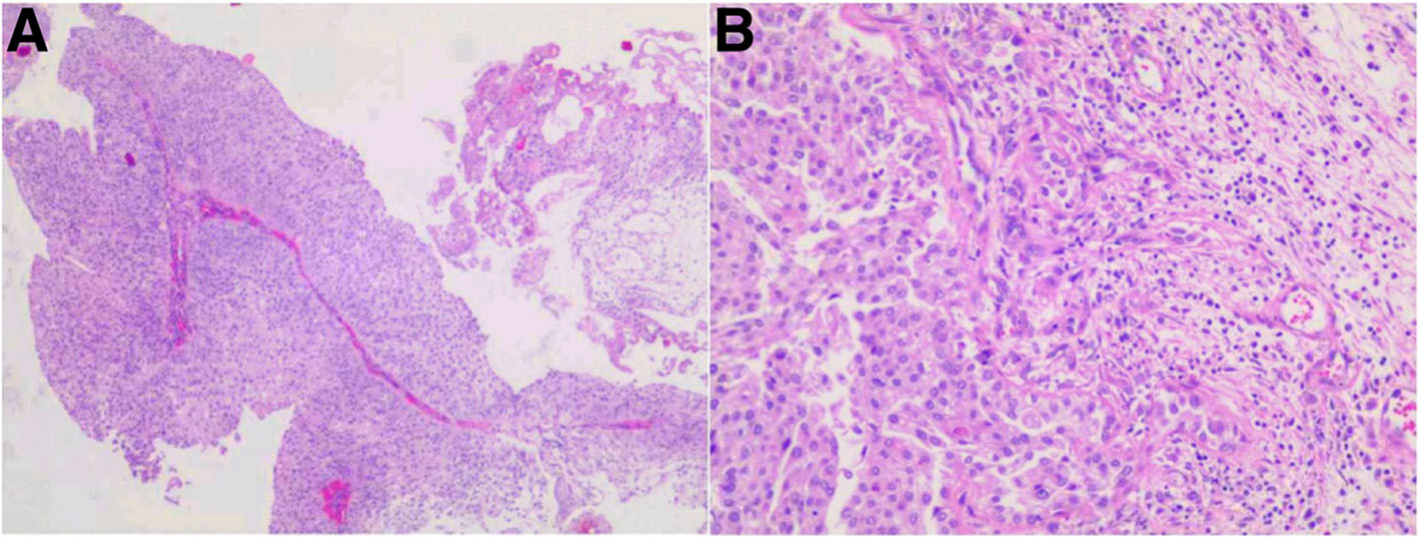
Postoperative pathological examination, low-power view of tumor of urethra. **a** Hematoxylin and eosin staining (magnification, × 40). **b** Hematoxylin and eosin staining (magnification, × 100)

Postoperative pathological examination: clinical data combined with microscopic findings and immunohistochemical results demonstrated high-grade UC (sarcoma-like variants) with extensive necrosis. Immunohistochemical results were PCK (AE1/AE3) (+), CK20 (−), CK7 (+), β-catenin (cell membrane +), vimentin (+), HMB-45 (−), Melan-A (−), and CD45 (LCA) (−) (Fig. 2).

One week after the operation, our patient took an infusion of intravesical chemotherapeutic drug (hydroxycamptothecin 40 mg), and refused further treatments. She left the hospital after being informed of the risks.

Treatment: on day 1 our patient was treated with blood test and catheterization. On day 2 she received an intravenous injection of cefoxitin sodium 0.5 g × 3/day. On day 4 she underwent cystoscopy. On day 10 she underwent bladder cancer diagnostic electrocision and continuous bladder irrigation. On day 18 she underwent intravesical chemotherapy with hydroxycamptothecin 40 mg.

After above treatments, her quality of life was greatly improved (including diet, sleep, stool, urine, and weight), and she had smooth urination without hematuria, urinary incontinence, or other symptoms. Briefly, her urination is smooth and there is no pain when urinating. Basically, she does not have nocturia, and has no symptoms such as frequency of urination, urgent urination, and dribble urination. She is of advanced age, her legs and feet are troublesome, and her quality of life has greatly improved, without frequent toilets, including sleep, diet, mood, and many other aspects. The potential risks are also reduced; for example, because she is old and has loose bones, with frequent urination she may fall and sustain fractures or even more serious conditions. Therefore, the consequences of frequent urination are incalculable. She did not continue to perform weekly intravesical chemotherapy for economic reasons. During the third to fourth months after discharge, she experienced recurrent urination and hematuria. No effective treatment was performed, and the tumor metastasized to her lungs after 1 year. She died of multiple organ dysfunction syndrome. She had no autopsy.

## Discussion

The disease in this article is very rare, therefore, the palliative resection combined with intravesical instillation of hydroxycamptothecin is extremely rare; it provides a prospective treatment for our patient’s disease, and is more economically and ethically convincing.

We acknowledge the limitations of the case, namely, retrospective analysis and it is an individual case. From 2013 to 2018, the First Clinical Hospital of Yichang received more than 10 patients with urethral carcinoma, excluding cases of metastatic cancer. Of these, only the case we reported here is PUC. This largely reflects the rarity of the disease. Since PUC is a rare tumor, the prognosis is of great significance. Although our patient survived only 1 year after diagnosis, her quality of life after treatment was greatly improved. For older people, it is an important thing to avoid going to the toilet often. It eliminates many hidden dangers and makes older persons feel happy. This is a butterfly effect process. Our patient did not continue treatments after discharge, which resulted in rapid deterioration and loss of life.

At present, the ratio of male versus female with PUC is 2.4:1 [4], and the high risk factors of males and females are different. PUC in male patients is mainly associated with urethral stricture, long-term repeated insertion of urethral catheter, radiation therapy, and STD (human papilloma, virus serotype 16). OUC in female patients is associated with factors such as urethral diverticulum, chronic infection of human papillomavirus, recurrent urinary tract infection, and other factors [11]. In male patients, clinical symptoms are mainly dysuria (41–48%), lower urinary tract irritation (20%), hematuria or urethral secretions (62%), urethral stricture (76%), urethra cutaneous fistula (10%), and abscess (5%) [12]. In female patients, most patients show urination irritation, urinary tract infection, and dyspareunia (70%), and the remaining minor manifestations were dysuria (23%) and hematuria (20%) [13]. In a physical examination, external genitalia and digital rectal examination are both basic and necessary. Female patients also need to undergo pelvic examinations. Examination of bilateral inguinal lymph nodes can provide evidence of disease progression. For the diagnosis of patients, the gold standard is cystourethroscopy with biopsy of bladder. It is more direct to observe the size, shape, and invasion scope of the tumor, whether the tumor is secondary to bladder tumor, and that can make a more definite diagnosis. Defining the nature of the disease requires staging and grading the disease. The prognosis of patients with PUC mainly depends on tumor and lymph node staging and the location of the primary tumor [14]. So the staging of the disease really matters. Its staging is mainly based on the tumor node metastasis (TNM) staging system; if the tumor occurs in the prostatic urethra the staging is different.

According to an epidemiological survey, survival rates of 5 and 10 years in patients with PUC in the USA were 46% and 29%, respectively, compared with 68% and 60% for overall cancer 5-year and 10-year survival rates [8]. The 1-year and 5-year survival rates in Europe are 71% and 54%, respectively [7]. The methods of treatment are varied, including surgery, radiotherapy, chemotherapy, and neoadjuvant chemotherapy. Monotherapy therapy is basically eliminated due to poor therapeutic effect and high recurrence rate, and multidisciplinary treatment has obvious advantages. When the TNM stage of a patient’s tumor is in the stage of Ta or Tis, the patient can undergo intravesical perfusion chemotherapy (once a week, a total of 6 weeks). In the past, the Bacillus Calmette–Guérin (BCG) vaccine was used, but now it is replaced by hydroxycamptothecin, platinum, and some other drugs because of side effects. The chemotherapeutic infusion drugs for different histologic types of cancer are also different. Now, intravesical perfusion chemotherapy is the dominant method that is performed before urethrectomy. Studies showed that 28–30% of patients have cancer recurrence after treatment [15, 16], which needs radical cystectomy, especially when the tumor invades the prostate duct or the muscular layer. Under this condition, pelvic lymph node dissection and urethral reconstruction surgery are needed. However, studies found that only 50% of patients have positive lymph nodes near the area of the iliac bone, therefore, it is necessary to consider carefully whether lymph node dissection is necessary [17]. For postoperative patients, local radiotherapy can also be used, but there are also some complications, such as urethral stricture, tissue necrosis, fistula, and edema. The sooner the cancer is discovered, the better the therapeutic effect. All in all, there are still many doubts about this disease, especially for advanced PUC; the optimal multidisciplinary treatment plan needs further confirmation in clinical practice.

Finally, with the passing of time, the application of immunohistochemistry in clinical practice is growing, its function is mainly: (1) diagnosis and identification of malignant tumors; (2) to determine the tissue source of malignant tumors; (3) to make the pathological type of a tumor more exact; (4) a small metastatic lesion or a remnant of the cancer cell is found; (5) to determine the prognosis of patients; and (6) to provide a treatment program for clinical use. Because of the limits of medical development, many diseases of immunohistochemistry are not studied thoroughly. A search of PubMed and Medline database did not find instances of the application of immunohistochemistry in PUC; no specific antigen has been found yet. The result of pathological biopsy in this patient is UC, the positive results of immunohistochemistry were PCK (AE1/AE3) (+), CK7 (+), β-catenin (cell membrane +), and vimentin (+). It was found that the expression of vimentin in urine cytology was helpful to distinguish reactive renal tubular cells and low-grade UC [18]. The expression of β-catenin in UC was significantly enhanced and involved in the occurrence and development of UC on the bladder urinary path [19]. Β-catenin overexpression may indicate that the UC is physiologically positive with poor prognosis and can be used as a molecular marker to facilitate targeted therapy development [20]. The CK20 immune response activity was most sensitive to the diagnosis of *in situ* carcinoma [21]. In combination with clinical data, endoscopic results, and immunohistochemical results, high-grade UC was supported. For a specific marker, current studies have found that NANOG and GATA3 are sensitive markers for UC, and might be a potential biomarker for early diagnosis of UC [22, 23]. However, further validation is needed in clinical practice, and it is believed that, in the near future, more convincing conclusions will be presented before us, and a PUC-specific marker will be found.

## Conclusions

Local surgical treatment and intravesical perfusion chemotherapy are a better treatment for elderly patients. Patients like us to try this treatment, but it also requires more prospective trials.

## Data Availability

Data reported in the article can be found in Yichang Central People’s Hospital’s electronic medical record system.

## Abbreviations

BCG: Bacillus Calmette–Guérin
CT: Computed tomography
EAU: European Association of Urology
MRI: Magnetic resonance imaging
PUC: Primary urethral cancer
STD: Sexually transmitted disease
TNM: Tumor node metastasis
UC: Urothelial carcinoma
